# The relationship between screen time before bedtime and behaviors of preschoolers with autism spectrum disorder and the mediating effects of sleep

**DOI:** 10.1186/s12888-023-05128-6

**Published:** 2023-08-30

**Authors:** Hanyu Dong, Tiantian Wang, Junyan Feng, Yang Xue, Feiyong Jia

**Affiliations:** Department of Developmental and Behavioral Pediatrics, The First Hospital of Jilin University, Changchun, 130021 China

**Keywords:** Screen time, Sleep, CSHQ, CBCL, Emotional/behavioral difficulties, Sedentary behavior

## Abstract

**Background:**

There are overlapping effects of screen time and sleep on children’s behavior. The purpose of this study was to explore the relationship of screen time with behavior problems in children with autism spectrum disorder (ASD) and the probable mediating effects of sleep, in order to provide evidence for the need for clinical identification and intervention.

**Methods:**

A sample of 358 preschoolers with ASD were included. We investigated the children’s basic characteristics of sex and age, ASD symptoms (ABC, CARS, and ADOS-2), neurodevelopment (GDS-C), sleep habits (CSHQ), and behavior (CBCL). Pearson correlation tests were used to determine the direct correlations among children’s screen time, CBCL, and CSHQ. Linear regression analysis was used to explore whether screen time predicted total score of CBCL. Multi-step linear regression analysis was used to investigate the mediating effect of sleep on the relationship between screen time and total score of CBCL.

**Results:**

Screen time before bedtime was correlated with CBCL and CSHQ, which indicated that screen time before bedtime was correlated with sleep and behavior in children with ASD. Screen time before bedtime was a predictor of CBCL total score (indicating children’s behavior), and CSHQ total score (indicating children’s sleep habits) played a partial mediating role between screen time before bedtime and children’s behavior.

**Conclusion:**

Clinicians should support and educate parents of children with ASD, which should focus on managing screen time, especially screen time before bedtime.

## Introduction

Autism Spectrum Disorder (ASD) is a neurodevelopmental disorder [1], and the latest epidemiological survey in the United States showed that the prevalence of ASD was 23.0 per 1,000 (1 in 44) among children aged 8 years old [2]. The main features are social communication dysfunction, restricted interests, and repetitive and stereotypical behaviors. In addition to the core symptoms, children with ASD often have additional emotional/behavioral problems, such as aggression, disruption, attention, anxiety, depression, and sleep problems [3]. The core symptoms of ASD and the emotional/behavioral problems can negatively impact children’s future social adjustment, academic achievement, occupational performance, and other domains [4]. The behavioral problems of children with ASD have gained much attention in recent years, and they are a major source of stress and worry for parents, although they are not a core feature of a child’s ASD diagnosis [5]. The behavioral problems are often socially unacceptable, can harm the child themself and others [6], and are in great need of medical recognition and intervention. Children with ASD are more likely to have behavioral problems than others [7, 8], and the proportion of children with ASD with at least one behavioral problem was reported to be 94% [7], which can severely limit their function.

Children are more sedentary than in the past, and screen time is an aspect of sedentary behavior [9]. Excessive screen time may affect many aspects of children, including emotional/behavioral problems [10–12]. The evidence for the effects of electronic screens on children without ASD is as follows. A 2020 meta-analysis of 19 studies suggested that typical developed (TD) children with high screen time were more likely to engage in violent behavior, including physical conflict, victimization, and bullying [13]. Exposure to media violence is one of the causal factors in real-life violence and aggression [14]. Furthermore, screen time at age 1–2 was associated with attention problems at age 7 [15]. Jackson found that TV viewing by toddlers was associated with later social/behavioral development in a longitudinal study in the USA [16]. Excessive use of electronic devices has also been linked to depression and increased potential suicide risk [17]. Séguin and Klimek found that the amount of TV watched increased the risk of aggression, anxiety, and hyperactivity between the ages of 3 and 5 [18], and similar findings were reported in even younger children [19]. A Chinese study found that screen time was associated with increased emotional/behavioral problems in children, including aggression, decreased prosocial behavior, and attention problems [20].

Concerningly, the current situation regarding screen time among children with ASD is more serious than that among other children [11]. Children with ASD are more attracted to electronic screens, spend more time with screens, and are more likely to develop symptoms of addiction [21]. Our previous study found that Chinese ASD children under 6 years of age spent an average of 3.34 h per day on electronic screens, which was much more than TD children (0.91 h per day) [22]. Healy also found that children with ASD had a longer screen time and less physical activity than TD children [23]. A study in Thailand also showed that children with ASD watched TV earlier and more frequently than TD children [24]. The average age when ASD children started watching TV was 6.44 months, and the average screen time was 4.60 h/d. The brains of children with ASD tend to be under-connected and less integrated [25], while screen time is associated with decreased global integration, decreased connectivity, and atrophy of gray matter in the frontal lobe [26], which may further aggravate the impact of electronic screens on children with ASD during a critical period of brain development [27].

Children with ASD often exhibit a variety of sleep problems [28, 29], such as decreased sleep duration, increased latency, and problems with wakefulness or nightmares, which may be associated with emotional/behavioral problems in these children. The prevalence of sleep problems in children with ASD is 40–80%, compared with 9–50% in TD children [30]. Some children may improve over time while others develop more serious sleep problems with age [31]. A Japanese study found that sleep problems in preschoolers with ASD were associated with behavioral problems [30], as assessed by the Child Behavior Checklist (CBCL). Sleep problems are associated with irritability, aggression, and self-injury in children with ASD [32–35]. A longitudinal study reported that sleep problems in children with ASD at age 2 predicted anxiety when they attended school [36].

Screen use may interfere with sleep by causing physiological arousal, melatonin production problems, and disturbed circadian rhythms in children [37, 38]. Given the adverse effects of screen use and sleep problems on behavior in children with ASD, a growing number of studies have explored the relationship between screen use and sleep problems. Screen use in children with ASD was found to be associated with sleep hygiene [39]. Christopher also found that, in boys with ASD, more time spent playing video games was associated with less sleep duration [40]. Among ASD children, greater daily screen time was associated with lower sleep duration, and older children and those from single-parent families were at higher risk of lower sleep duration [41]. Families of children with ASD may be more likely to use screen devices in an effort to calm children before bed [42]. So, naturally, studies have explored screen use before bedtime. A US study of school-age children and adolescents with ASD showed that children who were exposed to media with violent content before bedtime experienced significantly greater sleep onset delays and shorter overall sleep duration [38]. Having a TV or computer in the bedroom compared to not having these devices was associated with less sleep in boys with ASD [40]. Teenagers who used the Internet before bedtime were reported to sleep 51 min less than those who did not use the Internet before bedtime [43]. Studies estimated that the magnitude of the sleep delay associated with bedtime video gaming was up to 28 min [43, 44]. A study also reported that screen time before bedtime causes behavioral difficulties and conflict behaviors in children with neurodevelopmental disorders [45]. This suggests that screen time at specific times, not just total screen time, may affect children’s sleep.

There are overlapping effects of electronic screens and sleep on children’s behavior. In typically developed adolescents, reduced sleep and poorer sleep quality mediate the influence of screen time on attention problems, physical aggression, and emotional and somatic problems [46, 47]. Research has also explored the relationships between screen use, sleep, and behavioral problems in children with developmental disorders, pointing to the possible mediating role of sleep [11]. However, no studies have specifically examined the mediating effect of sleep on the relationship between screen time and behavior in children with ASD. We hypothesized that screen time in children with ASD is a predictor of behavioral problems, and sleep may play a mediating role. We conducted this study in order to explore our hypothesis and to provide evidence for the need for clinical identification and intervention for behavioral problems in children with ASD.

## Methods

### Participants

We recruited preschool children with ASD at the Department of Developmental Behavioral Pediatrics of the First Hospital of Jilin University. The recruitment started in January 2021 and was completed in March 2022. The final sample included 358 children, almost all of whom came from the northeast of China. Parents bring their children to the hospital on the recommendation of teachers, relatives, or primary care pediatricians, or they bring their children directly after recognizing their developmental problems (such as language and social interaction problems).

The inclusion criteria were as follows: children aged < 72 months, met the diagnostic criteria for ASD in the Diagnostic and Statistical Manual of Mental Disorders, Fifth Edition (DSM-5) and Autism Diagnostic Observation Schedule-2 (ADOS-2), and no prior systematic intervention or training program for the children and/or parents. The exclusion criteria were as follows: physical disability, clear metabolic or genetic disease, epilepsy, or any other conditions requiring the use of sedative hypnotics.

Ethics approval was granted by the ethics committee of our hospital. Informed consent was provided by the parents or caregivers of the children.

### Data collection

All assessments were conducted by experienced evaluators at our department, with strict quality control and standardized training. We collected data on the children’s demographic characteristics (sex and age) via evaluator interviews. Additionally, ASD symptoms were examined using the Autism Behavior Checklist (ABC), Childhood Autism Rating Scale (CARS), and ADOS-2. Neurodevelopment was evaluated using Griffiths Developmental Scales-Chinese (GDS-C). We also used the Children’s Sleep Habits Questionnaire (CSHQ) to evaluate sleep habits and CBCL to evaluate behavior.

The parents provided data on the children’s screen time. Parents filled out a screen time information form, which collected data on screen time per day on weekdays (min), screen time per day on weekends (min), screen time per day before bedtime (min), and age at first screen exposure (month). All the information was reported by the parents. We calculated the mean daily screen time as follows: mean daily screen time (min) = [screen time per day on weekdays (min) × 5 + screen time per day on weekends (min) × 2]/7. Total screen time per day included the screen time before bedtime. Screen time before bedtime was defined as the screen activity after children enter their bedroom or the screen exposure time before sleep.

The ABC is a 57-item screening checklist for autistic symptoms containing five subscales designed for parent interviews. The CARS consists of 15 subscales, each of which is scored on a continuum from normal to severely abnormal, which involved observation of the behavior of the children in a consulting room by experienced evaluators from our department. The reliability and validity of the ABC and CARS are sufficient [48]. The ADOS-2 [49], which is utilized as a diagnostic tool for ASD, is a semi-structured, standardized assessment tool that measures ASD symptoms in the domains of social relatedness, communication, play, and repetitive behaviors. It is considered the gold standard for ASD diagnostic evaluation. The ADOS-2 Modules 1 and 2 have calibrated severity scores: scores of 3 and 4 indicate low level evidence, scores of 5–7 indicate moderate-level evidence, and scores of 8–10 indicate high-level evidence. The ADOS-2 Toddler Module, which is used for children younger than 31 months, has no calibrated severity scores. GDS-C is widely used in the Chinese social context and has good reliability and validity [50]. It uses five independent subscales to assess the development level of children aged 0–2 years: locomotor (A scale), personal social skills (B scale), hearing–speech (C scale), hand–eye coordination (D scale), and performance (E scale). Children aged 3–8 years can also be assessed using the F scale to determine their practical reasoning. The GDS-C scores for the subscales are converted into development quotients (DQs; i.e., AQ, BQ, CQ, DQ, EQ, and FQ) as follows: DQ = DA × 100/CA, where DA is developmental age (with reference to the Chinese norm) and CA is chronological age [51].

CSHQ was developed in 2000 by Owens to evaluate sleep problems in children [52] and is widely used in children with ASD [53]. It includes 8 subscales (*bedtime resistance, sleep onset delay, sleep duration, sleep anxiety, night waking, parasomnias, sleep disordered breathing*, and *daytime sleepiness*), involving 33 items. Each item is rated 1 (occasionally) to 3 (usually), depending on how often it occurs. The higher the score, the more severe the sleep disorder [54]. It has good reliability and validity in China [55]. Parent-reported CSHQ has been reported to be an effective tool for assessing the overall sleep status of children with ASD [56].

CBCL was developed by Achenbach et al. [57]. The revised version of CBCL for 1.5- to 5-year-olds is widely used for behavioral and psychological screening, with good applicability in China [58]. It contains 99 problem items rated 0 = not true, 1 = somewhat or sometimes true, or 2 = very true or often true. The total score can be converted to a T score and compared with the Chinese norm to determine whether the child’s behavior is normal or abnormal. The higher the score, the more severe the behavior problem. The 7 clinical syndrome subscales are: *withdrawn, anxious/depressed, sleep problems, somatic complaints, aggressive behavior, attention problems*, and *emotionally reactive*. There are also 3 summary scales: *internalizing problems, externalizing problems*, and *total problems.*

### Statistical analysis

SPSS v23.0 (SPSS for Windows, IBM Corp., Armonk, NY, USA) was used to analyze the data. The continuous variables with normal distributions are presented as means ± standard deviations (SDs), and the categorical variables are presented as frequencies (percentages).

Descriptive statistics were used to describe the children’s demographic characteristics, electronic screen habits, ASD symptoms, and GDS-C scores (neurodevelopment). Pearson correlation analysis was used to determine the direct correlation between screen time, CBCL (behavior), and CSHQ (sleep). Linear regression analysis was used to explore whether screen time predicts total CBCL score. Multi-step linear regression analysis was used to investigate the mediating effect of sleep on the relationship between screen time before bedtime and total CBCL score. P < 0.05 was considered statistically significant.

## Results

The basic characteristics of all participants are presented in Table 1. A total of 358 children with ASD were enrolled, comprising 280 boys (78.2%) and 78 girls (21.8%), with a ratio of 3.59:1. The age of the children ranged from 24 to 72 months, with an average age of 42.20 ± 11.65 months.

**Table 1:** Basic characteristics of all participants

		N = 358
Sex	male(N,%)	280, 78.2%
	female(N,%)	78, 21.8%
Age (month) M(SD)		42.204(11.65)
Everyday screen time (min) M(SD)		94.77(110.02)
Screen time before bedtime (min) M(SD)		26.33(57.51)
Age of first screen exposure (month) M(SD)		12.77(7.79)
Griffiths M(SD)	physical mobility	68.19(16.10)
	personal social skills	50.78(17.63)
	hearing-speech	39.87(19.69)
	eye-hand coordination	55.89(18.65)
	performance	59.69(20.79)
	practical reasoning	71.06(21.89)
Total score of ABC M(SD)		55.44(16.72)
Total score of CARS M(SD)		34.51(4.38)
ADOS-2 severity scores^# M(SD)^		6.06 (1.621)

Children under 31 months of age use the M-T version of the ADOS, and there is no severity score. Therefore, 58 children had no severity scores, so no statistical analysis was performed

^#^Children who use the M-T and M-3 of the ADOS had no severity scores. Therefore, 58 children had no severity scores, so no statistical analysis was performed

The correlations between screen time, CBCL (behavior), and CSHQ (sleep) are shown in Table 2. Screen time per day had no relationship with CBCL total score or subscales, or CSHQ total score (all p values > 0.05). Screen time before bedtime had a positive correlation with *sleep problems* (r = 0.206, p = 0.001), *somatic complaints* (r = 0.167, p = 0.005) subscales and *total score of CBCL* (r = 0.149, p = 0.013). Screen time before bedtime also had a positive correlation with *sleep disordered breathing* (r = 0.183, p < 0.001), *parasomnias* (r = 0.136, p = 0.015) subscales and *total score of CSHQ* (r = 0.122, p = 0.030). There was a wide range of correlations among the total and subscale CSHQ and CBCL scores. *Total CSHQ score* was positively correlated with all subscales of CBCL except the *externalizing problems* subscale (see details of r and p values in Table 2). *Total CBCL score* was negatively correlated with *sleep onset delay* (r=-0.128, p = 0.023) and positively correlated with *parasomnias* (r = 0.232, p < 0.001), *sleep disordered breathing* (r = 0.200, p < 0.001), *daytime sleepiness* (r = 0.178, p = 0.002), and *total CSHQ score* (r = 0.248, p < 0.001). Other correlations among the subscales of CSHQ and CBCL are shown in Table 2.

**Table 2:** Correlation of screen time, behavioral problems and sleep (results of Pearson correlation)

	Everyday ST	ST before bedtime	Withdrawn	Anxious/depressed	Sleep problems	Somatic complaints	Aggressive behavior	Attention problems	Emotionally reactive	Internalizing	Externalizing	Total score of CBCL
Bedtime resistance	-0.092	0.025	0.042	0.186***	0.086	0.110*	-0.013	0.049	0.043	0.138*	0.037	0.075
Sleep onset delay	-0.104*	-0.057	-0.054	-0.019	-0.222***	-0.029	-0.160**	-0.005	-0.073	-0.031	-0.117*	-0.128*
Sleep duration	-0.006	-0.030	0.036	0.017	0.107	0.011	-0.039	0.093	-0.015	0.026	0.028	0.014
Sleep anxiety	0.000	0.048	0.046	0.212***	0.212***	0.085	-0.021	0.053	0.048	0.138*	-0.021	0.096
Night waking	0.031	0.092	0.039	0.144**	0.255***	0.111*	0.030	0.074	0.128*	0.096	0.017	0.140
Parasomnias	0.108*	0.136*	0.159**	0.183**	0.313***	0.128*	0.147**	0.137*	0.150**	0.202***	0.103	0.232***
Sleep disordered breathing	0.095	0.183***	0.067	0.179**	0.159**	0.198***	0.159**	0.185**	0.109	0.149**	0.100	0.200***
Daytime sleepiness	-0.019	0.074	0.130*	0.131*	0.123*	0.118*	0.149**	0.089	0.122*	0.151**	0.050	0.178**
Total score CSHQ	0.011	0.122*	0.146**	0.261***	0.296***	0.198***	0.121*	0.182**	0.159**	0.238***	0.094	0.248***
Everyday screen time	/	0.295***	0.021	-0.031	0.090	0.047	-0.007	-0.034	-0.033	-0.009	0.021	0.012
Screen time before bedtime	0.295***	/	0.085	0.084	0.206**	0.167**	0.114	0.063	-0.012	0.094	0.058	0.149*

*P<0.05

**P<0.01

***P<0.001

The linear regression analysis indicated that screen time before bedtime was a predictor of total CBCL score (t = 2.143, p = 0.033), while total daily screen time did not predict total CBCL score (t = 0.726, p = 0.469) (Table 3). The longer the screen time before bedtime, the higher the total CBCL score.

**Table 3:** Regression analysis suggested that electronic screen time before bedtime was a predictor of children’s behavior

	B(SE)	β	t	P	R^2^
Everyday screen time	0.010(0.014)	0.045	0.726	0.469	0.024
Screen time before bedtime	0.051(0.024)	0.134	2.143	0.033*	

**Fig. 1:**
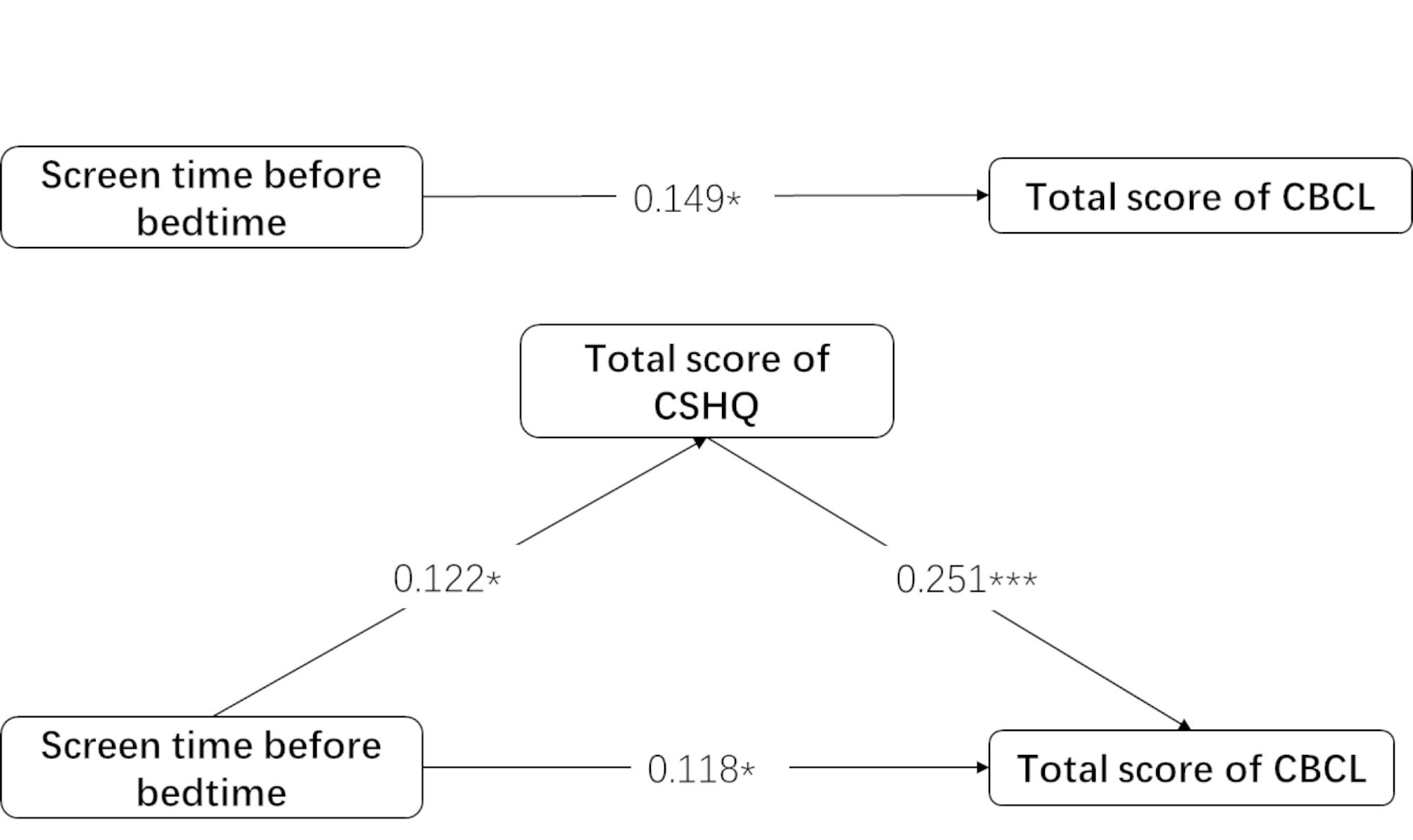
Coefficient of determination of relationship between screen time before bedtime and total score of CBCL mediated by sleep

The multi-step linear regression analysis indicated the mediating role of total CSHQ score in the relationship between screen time before bedtime and total CBCL score (Table 4; Fig. 1). The direct effect of screen time before bedtime on total CBCL score was significant (p = 0.013). The indirect effect was also significant (p = 0.043). The mediating effect model showed that screen time before bedtime significantly positively predicted total CSHQ score, with an explanation rate of 1.5%. Together, screen time before bedtime and total CSHQ score significantly positively predicted total CBCL score, with an explanation rate of 8.4%. In summary, total CSHQ score partially mediated the relationship between screen time before bedtime and total CBCL score.

**Table 4:** Sleep mediates the relationship between screen time before bedtime and total CBCL scores

	total score of CBCL (Step 1)				total score of CSHQ (Step 2)				total score of CBCL (Step 3)			
	B(SE)	β	P	t	B(SE)	β	P	t	B(SE)	β	P	t
screen time before bedtime	0.056(0.022)	0.149	0.013*	2.509	0.014(0.006)	0.122	0.030*	2.181	0.045(0.022)	0.118	0.043*	2.035
total score of CSHQ									0.914(0.210)	0.251	<0.001***	4.347
F	6.295				4.757				12.798			
R^2^	0.022				0.015				0.084			

## Discussion

This is the first study, to our knowledge, to examine the relationships between screen time before bedtime, sleep, and behavior in children with ASD. The main findings of our research on children with ASD were: [1] screen time before bedtime is correlated with both behavior and sleep, although this is not totally consistent with our hypothesis, and [2] screen time before bedtime is a predictor of total CBCL score, with sleep playing a partial mediating role in this relationship.

### The relationship between screen time before bedtime and CBCL

We found that, in children with ASD, total daily screen time did not predict the total score of CBCL, but screen time before bedtime was an independent predictor. This is not consistent with our hypothesis. The results obtained by several previous studies are also not completely consistent, but a greater number of studies support our results. A study of typically developing children ages 8 to 17 found that screen time before bedtime was strongly associated with sleep behavior, but not significantly associated with inattention [59]. In contrast, Hysing et al. concluded that using electronic devices for an hour before bedtime did not affect hyperactivity symptoms in teenagers [60]. This may have been because the participants in the study were 16–19 years old, and their situation may be more related to that of adults rather than preschoolers. A large study of elementary school children in Japan reported that screen time before bedtime affected children (regarding obesity, physical activity, dry eyes, and learning ability) more than no screen time before bedtime [61], which was consistent with our results. Screen time before bedtime can lead to daytime sleepiness, fatigue, and behavioral problems. A review of the effects of nighttime media exposure on adolescents showed relationships between excessive use of electronic media at night and adolescent behavior [62].

However, much of the evidence focuses on [1] typically developing children and [2] school-aged children and adolescents, with adverse effects of bedtime screen time in both groups. The brain development characteristics of children with ASD make them more likely to be affected by adverse environments [63], and the brain development of young children is immature, so children with developmental disabilities and young children may have more serious adverse effects [64]. Along with the effect of sleep as an intermediary factor, these effects involve the direct effect of electronic screens, the mechanism of which is unclear. There are animal studies on the neurodevelopmental effects of exposure to radiofrequency radiation from electronic products, but further research is needed [65].

### The partial mediating role of CSHQ score between screen time before bedtime and CBCL score

We found that sleep played a role as a partial mediating factor in the relationship between screen time and behavior. Although there are few studies that directly address children’s screen time before bedtime, sleep, and behavior together to support our conclusions, there is a lot of circumstantial evidence. Lin et al. showed that sleep problems completely mediated the relationship between the number of screen devices in the bedroom and emotional/behavioral problems in children with neurodevelopmental disorders [11]. However, only 39.5% of the subjects in this study were children with ASD. Tong et al. showed that bedtime screen time moderated the relationship between hyperactivity/attention and sleep [66], but this research focused on children with ADHD rather than ASD. Beyens et al. showed that increased evening TV viewing among 3- to 5-year-old children was associated with poorer sleep consolidation, suggesting less mature sleep patterns [67]. Garrison et al. concluded that media negatively affects children’s sleep, especially in the context of evening use or having a TV in the child’s bedroom [68]. The negative consequences of screen use on sleep are increased by longer duration of use and greater proximity to bedtime.

A possible reason for the effect of electronic screens on sleep is the blue light hypothesis, which states that short-wavelength-enriched light emitted by electronic media suppresses the production of melatonin in the brain [37, 69], which deregulates circadian rhythms [70] and results in sleep problems. Increased psycho-physical arousal caused by media content or decreased sleep quality due to excessive light exposure may also cause sleep problems [38]. Electromagnetic radiation emitted by electronic screens may alter melatonin release by affecting the signal transduction pathway leading from the norepinephrine receptor to N-acetyltransferase and hydroxyl-indole-O methyltransferase [71]. Sleep problems lead to insufficient sleep at night among children; this leads to more naps and is also indicative of an immature sleep pattern, which is detrimental to child development [67]. However, a study showed that typically developing children playing games on electronic devices for an hour before bedtime did not have a reduced sleep duration, while children with ASD did [40]. It may be reasonable to speculate that abnormalities in brain development in children with developmental disabilities account for this difference.

In typically developing children, sleep issues are associated with a wide range of behaviors, including both internalizing and externalizing symptoms [72], and sleep issues can also affect attention and school performance [73]. However, due to the behavioral characteristics of children with ASD, sleep may have even larger effects on their behavior. Short sleep duration in children with ASD is positively correlated with increased stereotypic behavior [74], and also aggression, irritability, and other emotional problems [31, 74, 75]. The findings in this study support previous research on the correlation of sleep habits with behavior. One explanation for the occurrence of sleep abnormalities in children with ASD is that there are molecular and cellular abnormalities in brain development in children with ASD, and abnormal sleep itself can be one of many clinical manifestations [76]. Screen time may be an environmental factor that adversely affects sleep and further affects brain development in children with ASD. This may create a vicious cycle in these children. There are two hypotheses regarding how sleep affects children’s behavior [77]: [1] insufficient sleep prevents or reduces brain activities required for brain maturation, affect regulation, and learning and [2] insufficient sleep increases daytime sleepiness and reduces alertness, which can hinder daytime functioning. These hypotheses indicate that sufficient sleep appears to be an important predictor of children’s psychological well-being and behavior [78].

It is also important to note that our results suggest that the relationships among screen time before bedtime, sleep, and behavior were not strong. The explanatory rate of the model was not high. This indicates that many factors may influence the behavior of children with ASD (although we focused on the mediating role of sleep). This may be a limitation of this study, and it provides directions for future research. And also, considering the cross-sectional nature of this study, we should treat the results of this study with caution. The causal relationship among screen time, sleep, and behavior was unclear. Thus, future research should focus on interventions reducing screens before bedtime for children with ASD, and increasing clinician, parents, and teachers’ awareness of the potential impact of screens on sleep and behavior.

### Limitations

Studies found that violent content and highly stimulating content on electronic screens before bedtime can affect children’s sleep [68, 79] and screen types (e.g., tablets, computers, smartphones, and TVs) and content (e.g., education, video games, cartoons, and movies) are related to children’s problem behaviors [78], but we did not focus on the content of electronic screens before bedtime. Also, without assessing screen content, it is impossible to know whether parents are using electronic screens before bedtime for the purpose of promoting sleep (like some apps for stories or songs). This limitation of the study provides a direction for future research. In the future, we plan to categorize electronic screen content (e.g., education, video games, and violent content) in detail.

Secondly, as previously mentioned, this study is a cross-sectional study, and cannot be concluded the causal relationship among screen time, sleep and behavior. And some children with ASD are used to using screens before bedtime, they may have a learned association to fall asleep only with the sound and light from screens. In this case, removing screens before bed may actually increase behavior and sleep problems in the short term. So, we would be cautious in advising parents to manage screen time before bedtime (further research is needed to support this). Meanwhile behavioral interventions to improve sleep skills should be considered in children with ASD who had high screen use before bed.

Additionally, as many factors influence the behavior of children with ASD (although we focused on the mediating role of sleep), in future clinical work, we need to identify the other mediating factors to better explain the relationship between screen time and behavior in children with ASD.

Lastly, data collection in this study relied on parent ratings, which is a major limitation. The information is subjective, and there may have been recall bias. It is hoped that data can be captured in a more objective way, such as by using family camera information collection and sleep monitoring, in the future.

## Conclusion

Screen time before bedtime is a predictor of behavior among preschoolers with ASD, and the relationship is mediated by sleep habits. It is important to increasing clinician, parents, and teachers’ awareness of the potential impact of screens on sleep and behavior.

## Data Availability

The datasets used and analyzed during the current study are available from the corresponding author on reasonable request.

## List of abbreviations

ABC: Autism Behavior Checklist
ADOS-2: Autism Diagnostic Observation Schedule-2
ASD: Autism Spectrum Disorder
CARS: Childhood Autism Rating Scale
CBCL: Child Behavior Checklist
CSHQ: Children’s Sleep Habits Questionnaire
DSM-5: Diagnostic and Statistical Manual of Mental Disorders
GDS-C: Griffiths Development Scales-Chinese
